# MCPIP1 contributes to clear cell renal cell carcinomas development

**DOI:** 10.1007/s10456-017-9540-2

**Published:** 2017-02-14

**Authors:** Janusz Ligeza, Paulina Marona, Natalia Gach, Barbara Lipert, Katarzyna Miekus, Waclaw Wilk, Janusz Jaszczynski, Andrzej Stelmach, Agnieszka Loboda, Jozef Dulak, Wojciech Branicki, Janusz Rys, Jolanta Jura

**Affiliations:** 1 Department of General Biochemistry, Faculty of Biochemistry, Biophysics and Biotechnology, Jagiellonian University, 7 Gronostajowa St, 30-387 Krakow, Poland; 2 Center of Oncology, Maria Skłodowska-Curie Memorial Institute, Garncarska 11, 31-115 Krakow, Poland; 3 Department of Medical Biotechnology, Faculty of Biochemistry, Biophysics and Biotechnology, Jagiellonian University, Krakow, Poland; 4 Malopolska Centre of Biotechnology, Jagiellonian University, Krakow, Poland

**Keywords:** Clear cell renal cell carcinoma (ccRCC), MCPIP1 (Regnase-1), HIF1α, HIF2α, Angiogenesis

## Abstract

*Monocyte Chemoattractant* protein-induced protein 1 (*MCPIP1*), also known as Regnase-1, is encoded by the *ZC3H12a* gene, and it mediates inflammatory processes by regulating the stability of transcripts coding for proinflammatory cytokines and controlling activity of transcription factors, such as NF-κB and AP1. We found that MCPIP1 transcript and protein levels are strongly downregulated in clear cell renal cell carcinoma (ccRCC) samples, which were derived from patients surgically treated for renal cancer compared to surrounded normal tissues. Using Caki-1 cells as a model, we analyzed the role of MCPIP1 in cancer development. We showed that MCPIP1 expression depends on the proteasome activity; however, hypoxia and hypoxia inducible factor 2 alfa (HIF2α) are key factors lowering MCPIP1 expression. Furthermore, we found that MCPIP1 negatively regulates HIF1α and HIF2α levels and in the case of the last one, the mechanism is based on the regulation of the half time of transcript coding for HIF2α. Enhanced expression of MCPIP1 in Caki-1 cells results in a downregulation of transcripts encoding VEGFA, GLUT1, and IL-6. Furthermore, MCPIP1 decreases the activity of mTOR and protein kinase B (Akt) in normoxic conditions. Taken together, MCPIP1 contributes to the ccRCC development.

**Electronic supplementary material:**

The online version of this article (doi:10.1007/s10456-017-9540-2) contains supplementary material, which is available to authorized users.

## Introduction

Kidney cancer is among the ten most common cancers in both men and women. More than 90% of kidney cancers originate from epithelial cells and are referred to as renal cell carcinoma (RCC). The most frequent form of this type of cancer is clear cell renal cell carcinoma (ccRCC), encompassing 75% of all renal cell carcinomas [1].

In more than 90% of ccRCC samples, the loss of short arm chromosome 3 is observed [2]. This deletion includes the locus for the von Hippel–Lindau (VHL) tumor suppressor gene, responsible for regulating the stability of hypoxia inducible factors, HIF-1α and HIF-2α. The VHL protein forms a stable complex with other proteins, possessing E3 ubiquitin ligase activity. This complex is responsible for targeting HIFs for polyubiquitination and subsequent proteasomal degradation [3]. Under hypoxic conditions, or when the VHL gene is mutated or lost, HIFs accumulate, leading to increased expression of genes encoding vascular endothelial growth factor (VEGFA), platelet derived growth factor (PDGF), transforming growth factor-α (TGFα), erythropoietin (EPO), as well as receptors potentially important in RCC oncogenesis [4]. Further studies have shown that other genes in the short arm of chromosome 3, which are responsible for the chromatin remodeling and epigenetic modifications, are crucial for ccRCC development [5–7].

Inflammatory pathways have been extensively studied in ccRCC; however, data concerning the role of proteins that regulate stability of transcripts coding for proinflammatory mediators are limited. In our previous study, we analyzed the role of *Monocyte Chemoattractant* protein-induced protein 1 (*MCPIP1*), encoded by the *ZC3H12a* gene. MCPIP1 (also known as Regnase-1) possesses the N terminus of the PilT protein (PilT N terminus or PIN domain), which has RNase properties and regulates half time of transcripts coding for certain proinflammatory cytokines including: IL-1β [8], IL-2 [9] or IL-6 [10]. Moreover, MCPIP1 also suppresses microRNA biosynthesis via cleavage of the terminal loops of precursor miRNAs, counteracting Dicer, a central ribonuclease in miRNA processing [11]. Besides well-documented RNAse properties, MCPIP1 is considered a negative regulator of the NF-кB signaling pathway [12, 13].

In the present study, we hypothesized a role of MCPIP1 in the etiology of ccRCC. To this purpose, we analyzed ccRCC samples and adjacent normal tissues from patients surgically treated for renal cancer to estimate the level of transcripts coding for MCPIP1. Additionally, we determined correlations between MCPIP1 mRNA levels and transcripts coding for other proteins important for ccRCC development and invasiveness. To clarify the impact of MCPIP1 on ccRCC biology, we used ccRCC cell lines and assessed the potential mechanistic role of MCPIP1 in ccRCC development and examined the possible usefulness of this protein as a therapeutic target in ccRCC therapy.

## Results

### Characteristics of ccRCC and non-tumor tissues from patients

The data describing patients and tumor tissues are presented in Table 1. Non-tumor and ccRCC tissues were analyzed from 47 patients, with 42.86% represented by females. The mean age of patients at surgery was 62.5 (range 33–83). The tumor grades, according to Fuhrman, were as follows: G1-14.9%, G2-34.0%, G3-31.9%, and G4-19.2%. Twenty-seven patients (57.4%) had tumor limited to kidney, while the remainder (42.6%) had invasion into major veins. Eighteen patients (38.3%) had aneuploid nuclei (samples with Fuhrman grade 3 and 4).

**Table 1:** Patients characteristics

Patients	
*N* (%)	47 (100)
Age (years)	
Median (range)	62.5 (33–83)
Sex—*N* (%)	
Male	27 (57.45)
Female	20 (42.55)
Histologic grade (Fuhrmann) *N* (%)	
Grade I	7 (14.89)
Grade II	16 (34.04)
Grade III	15 (31.91)
Grade IV	9 (19.15)
Invasion—*N* (%)	
Limited to the kidney	27 (57.45)
Invasion into major veins	20 (42.55)
Tumor size (cm)	
Median (range)	4.5 (1.4–16.0)
Proliferation index (S + G2/M) [%]	
Median (range)	4.15 (0.6–48.4)
Ploidy—*N* (%)	
Diploid	29 (61.70)
Aneuploid	18 (38.3)

**Fig. 1:**
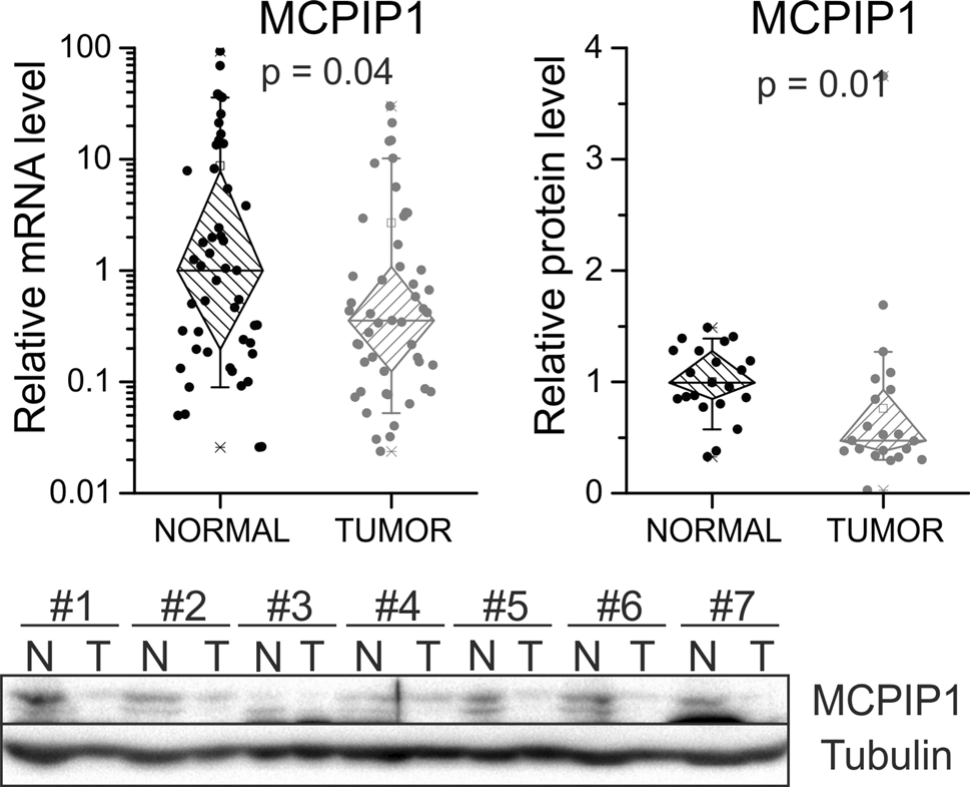
Transcript and protein level of MCPIP1 in ccRCC tissue samples. mRNA levels were analyzed using qRT-PCR for 47 patients in ccRCC tissue (referred as tumor) and non-neoplastic kidney tissue (normal). For each sample, transcript levels were normalized to the reference gene (PPIA—peptidylprolyl isomerase A) expression level. The mRNA level for non-tumor samples was set to 1. The MCPIP1 protein level was analyzed by western blot from 21 tumor samples and adjacent normal tissues. Then, densitometry was performed. Tubulin and GAPDH were used as a loading control and tubulin was used as a reference. The *p* values were estimated using Mann–Whitney (Wilcoxon) *W* test

In the case of *MCPIP1* transcript, we observed statistically significant downregulation of gene expression in 47 ccRCC samples in comparison with non-tumor tissues. Similarly, we noticed also diminished level of MCPIP1 protein in 21 ccRCC samples in comparison with corresponding normal tissues (Fig. 1).

### Caki-1 as a model to study ccRCC

**Fig. 2:**
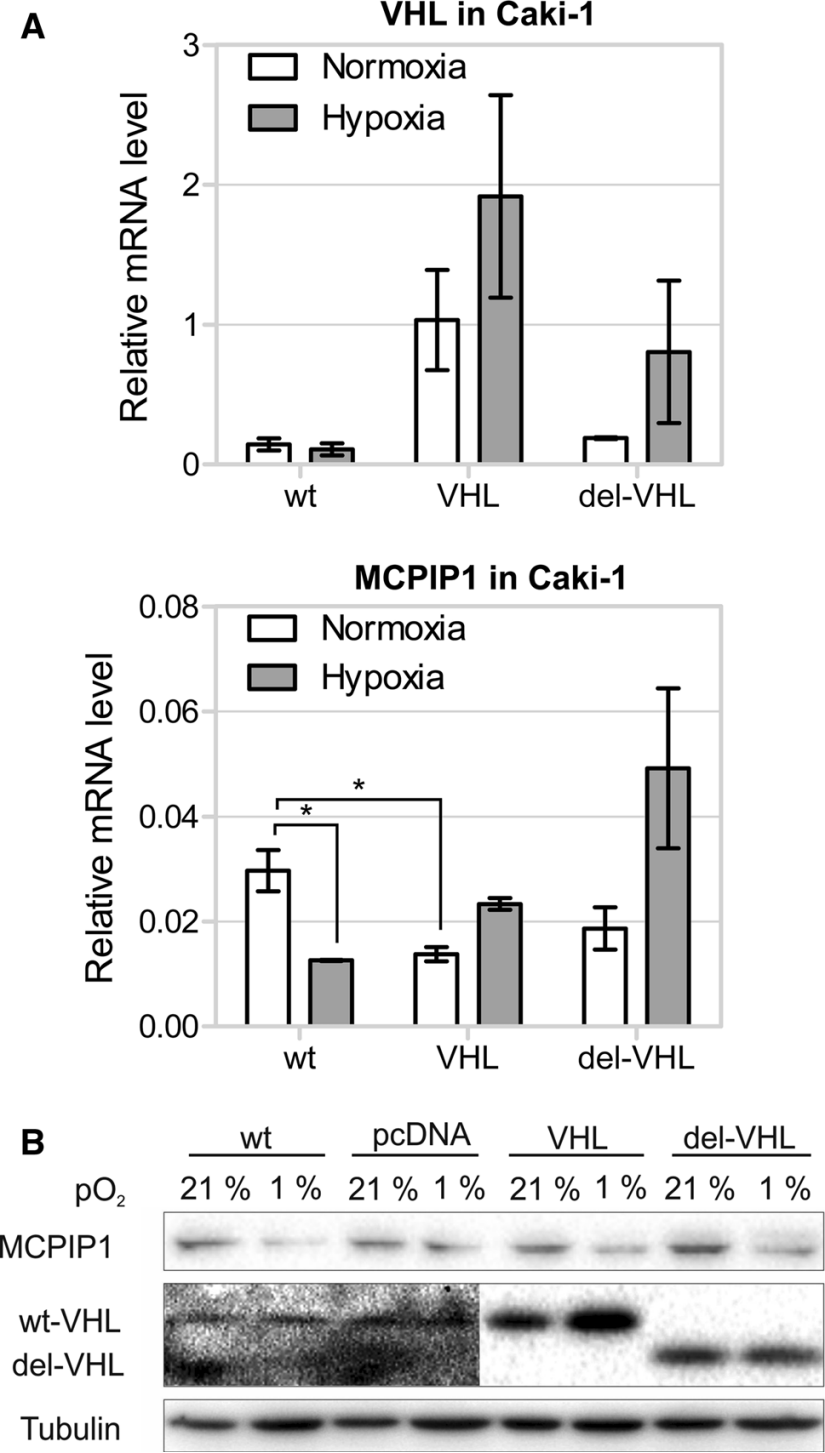
Overexpression pVHL in Caki-1 cells. **a** Cells were transfected with plasmid HA-VHL-pRc/CMV containing wild-type VHL gene (VHL) and a mutant form of VHL (deletion of C-terminal domain, del-VHL and non-treated cells (WT) as a control. Cells were cultured under hypoxic and normoxic conditions for 24 h. RNA level was estimated by Q-RT-PCR with specific primers for mentioned genes. Q-RT-PCR results are presented as mean ± SEM of three independent experiments. The *p* values were estimated using ANOVA with post hoc Bonferroni test (*p < 0.05). **b** Cells were transfected with plasmid HA-VHL-pRc/CMV containing wild-type VHL gene (VHL) and a mutant form of VHL (deletion of C-terminal domain, del-VHL). As control, non-treated cells (NT) and cells transfected with empty pcDNA3 plasmid (pcDNA) were used. After 24 h Western blots were performed with antibodies specific for MCPIP1, VHL, and α-tubulin

To study the role of MCPIP1 in clear cell renal cell carcinoma etiology, the ccRCC cell line, Caki-1, was used. These cells display epithelial morphology of ccRCC and possess an active form of VHL. To clarify, whether VHL plays a direct role in the regulation of MCPIP1 levels, Caki-1 cells were transfected with a vector expressing wild-type VHL or its mutant form (with a deletion of the C-terminal domain) [14]. Overexpression of wild-type VHL or its mutant form did not influence MCPIP1 transcript and protein levels in normoxic or hypoxic conditions (Fig. 2).

To further assess the role of VHL in MCPIP1 regulation, we utilized A498 cell line, which possess completely disrupted VHL function. First, we compared both cell lines on the molecular level. As shown in Suppl. Fig. 1a, the VHL transcript level is much higher in Caki-1 cells than in A498, but protein is only detected in Caki-1. Both cell lines express similar levels of mRNA coding for HIF1α, but HIF1α protein was visualized by western blot only in Caki-1 cells. HIF2α expression was detectable in both cell lines at the mRNA and protein levels; however, the level of HIF2α was higher in A498 cells, probably due to inactive VHL [14] (Suppl. Fig. 1). Both cell lines differed in the level of MCPIP1 transcript and protein. In Caki-1 cells, twofold higher transcript levels were observed in comparison with A498 cells, but the reverse situation was seen in case of protein. Caki-1 cells expressed significantly lower levels of protein than did A498 cells (Suppl. Fig. 1). Moreover, the level of the transcript coding for glucose transporter (GLUT1) and *VEGFA* was lower in Caki-1 than in A498 cells (Suppl. Fig. 1).

Overexpression of either wild-type VHL or its mutant form in the A498 cell line did not influence the level of MCPIP1; thus, the regulation of MCPIP1 is VHL independent. As a positive control for VHL activity, a diminished level of transcripts coding for VEGFA and GLUT1 was observed. In the mutant form of VHL, the decrease was not observed for either transcript. Therefore, in active form of VHL, HIFs are degraded, and there is no expression of transcripts coding for VEGFA and GLUT1 (Suppl. Fig. 2).

### Hypoxia decreases the level of MCPIP1

**Fig. 3:**
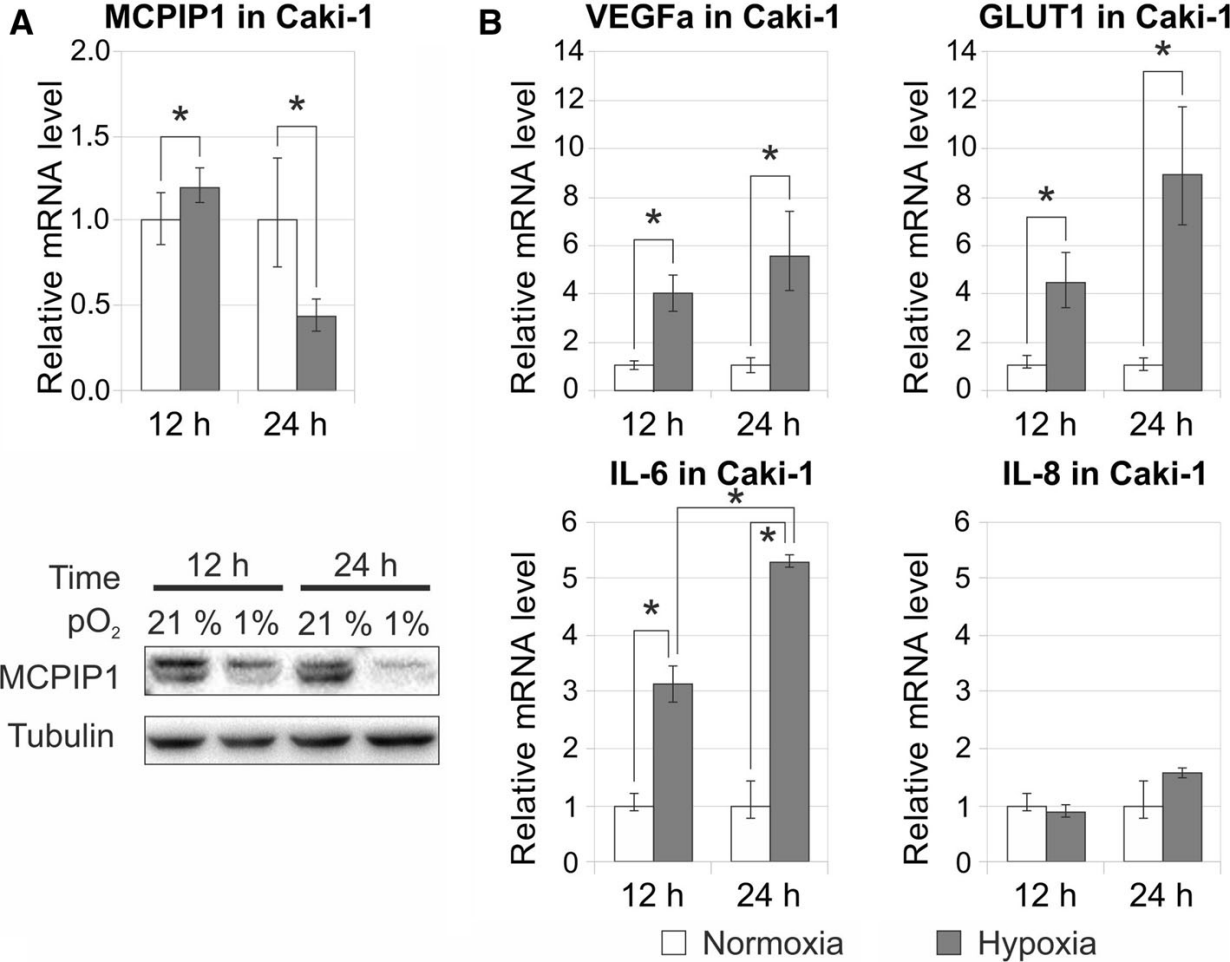
Influence of hypoxic condition on transcript and protein expression of MCPIP1 (**a**) and on expression of transcript coding for VEGFA, GLUT1, IL-6, and IL-8 (B) in Caki-1. Cells were cultured under hypoxic and normoxic conditions for 12 and 24 h. RNA level was estimated by Q-RT-PCR with specific primers for mentioned genes. Q-RT-PCR results are presented as mean ± SEM of three independent experiments. For each sample, the transcript level was normalized to reference gene (RPS13) expression level. The mRNA level for Caki-1 cells in normoxia was set to 1. The *p* values were estimated using ANOVA followed by Tukey’s HSD test (*p < 0.05). Protein level was analyzed by western blot with specific antibodies for MCPIP1 and α-tubulin. Presented western blot data are representative of three independent experiments

Hypoxia is an important factor in the development of several types of malignancies acting as a modulator of the mTOR pathway through the accumulation of HIF1α [15]. Consequently, many genes that control tumor biology are regulated by oxygen. Thus, we started our study analyzing the effect of oxygen supply efficiency on expression of selected genes. Caki-1 cells were exposed to normoxia (21% O_2_) or hypoxia (1% O_2_) for 12 and 24 h. Low oxygen supply caused a decrease in MCPIP1 protein level after 12 h, and the effect was more prominent after 24 h. Lower level of protein during hypoxia correlated with reduced MCPIP1 transcript level after 24 h of oxygen depletion in comparison with normoxic conditions (Fig. 3a). As previously shown in other cell types [16–18], mRNA levels for VEGFA and GLUT-1 were upregulated by hypoxia in Caki-1 cells (Fig. 3b). We noted that IL-6 expression was also upregulated by hypoxia after 12 h and the effect was even more meaningful after 24 h. In contrast, no changes were observed for mRNA encoding IL-8 (Fig. 3b). Thus, hypoxia negatively regulates MCPIP1 transcript and protein expression, but at the same time, transcripts coding for VEGFA, GLUT1, and IL-6 was upregulated. Diminished level of MCPIP1 during hypoxia is observed in cells with active form of VHL: Caki-1 and Caki-2 (both ccRCC cell lines), and with inactive form of VHL: A498 cells (ccRCC cell line). Furthermore, hypoxia downregulated MCPIP1 in embryonic kidney cell line (HEK293) and an immortalized proximal tubule epithelial cell line (HK2) (Suppl. Fig. 3A-C).

### Increased MCPIP1 level following MG132 treatment is the result of *de novo* expression

**Fig. 4:**
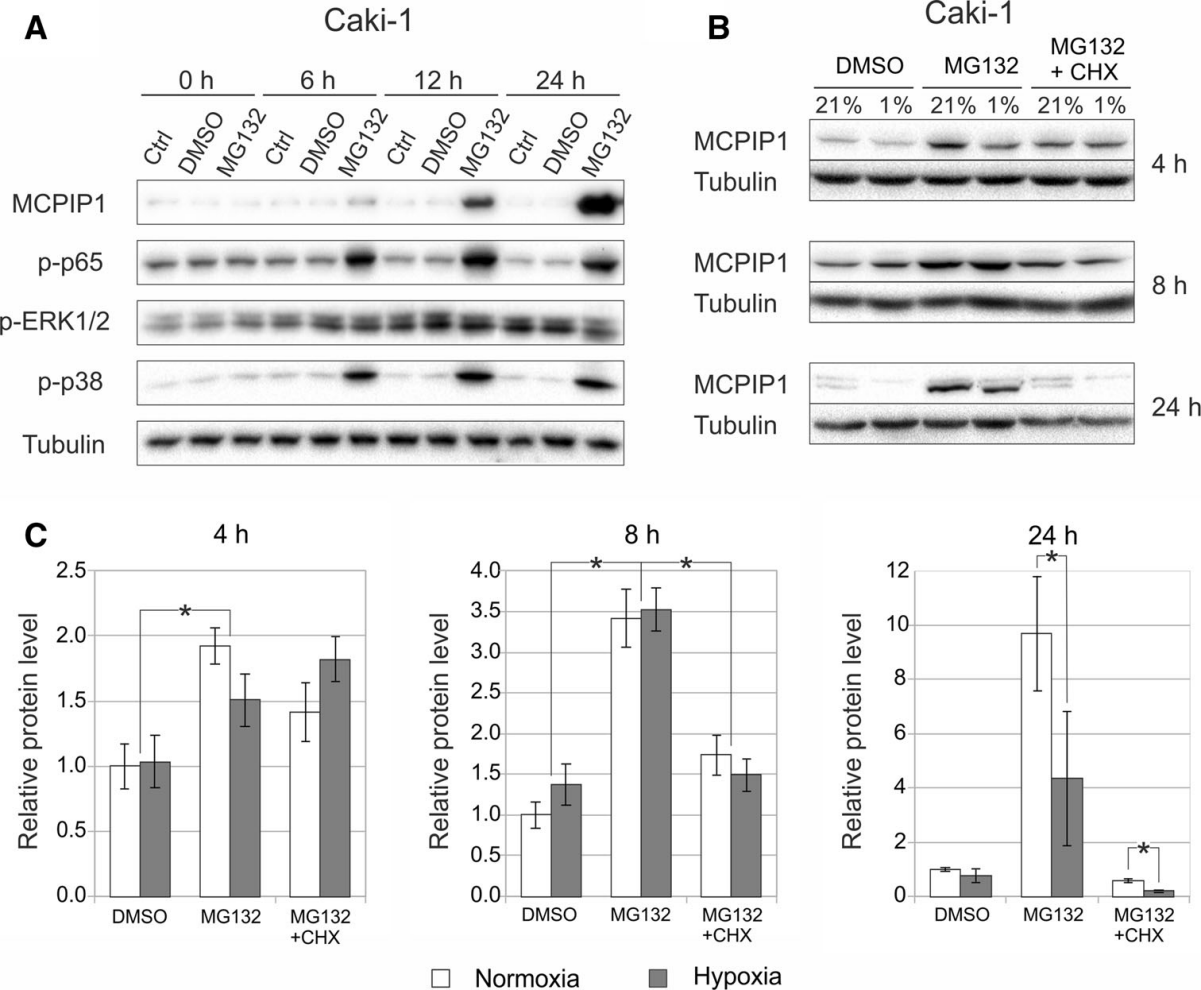
Proteasome inhibitor MG132 increases the expression of MCPIP1 and activates NF-κB (p65), and p38. **a** Caki-1 cells were treated with 1 µM MG132 or DMSO (control) for the indicated time periods. Protein extracts were subjected to western blot analysis with antibodies specific for MCPIP1 and phosphorylated signaling proteins (p65, Akt and p38). α-Tubulin was used as loading control. *Blots* are representative of three independent experiments. **b** Influence of MG132 and MG132 + cycloheximide (CHX) treatment on MCPIP1 expression in normoxia and hypoxia was analyzed by western blot. Graphs show mean ± SEM of three independent experiments (**c**). Statistical analysis was performed with ANOVA followed by Tukey’s HSD test (*p < 0.05)

As we previously established, HepG2 and HeLa cells treated with proteasome inhibitor MG132 display accumulation of MCPIP1. Moreover, we presented evidences that *de novo* mRNA synthesis is involved in the increase of MCPIP1 protein following MG132 treatment [19, 20]. Thus, we posed the question whether the same mechanism of MCPIP1 regulation is present in Caki-1 cells. After a 12-h incubation of these cells with 1 μM MG132 in normoxic conditions, the level of MCPIP1 increased, and after 24 h, the increase was even more prominent. The strong accumulation of MCPIP1 was accompanied by robust phosphorylation of p65 subunit of NF-κB, and p38 MAP kinase, while the phosphorylation status of ERK1/2 kinase was not changed (Fig. 4a).

As MCPIP1 levels are dependent on proteasome activity in normoxia, it was also interesting to see whether such phenomena takes place in cells cultured under low oxygen conditions. Caki-1 cells were treated with MG132 and cultured in both normoxia and hypoxia for 4, 8, and 24 h. Compared to control cells (DMSO treated cells), we observed an accumulation of MCPIP1 after MG132 stimulation, in both normoxia, and hypoxia; however, the effect was not so prominent in cells kept 24 h in low oxygen concentration (Fig. 4b, c).

To determine, whether accumulation of MCPIP1, after inhibition of proteasome, is the result of *de novo* synthesis, cycloheximide (CHX, 5 µg/ml) was added to cells treated with MG132 for 4, 8 and 24 h. The addition of CHX to cells treated with MG132 resulted in a strong decrease of MCPIP1 protein level in cells cultured in normoxia after 8 h, and even more prominently after 24 h. The effect was especially strong in cells cultured in hypoxic conditions for 24 h in comparison with cells treated only with MG132. This experiment showed that accumulation of MCPIP1 in Caki-1 cells depends on *de novo* synthesis as inhibition of translation with CHX inhibited MCPIP1 protein increase following MG132 treatment and the effect is especially meaningful in hypoxic conditions (Fig. 4b, c).

### HIF2α negatively regulates MCPIP1 levels

**Fig. 5:**
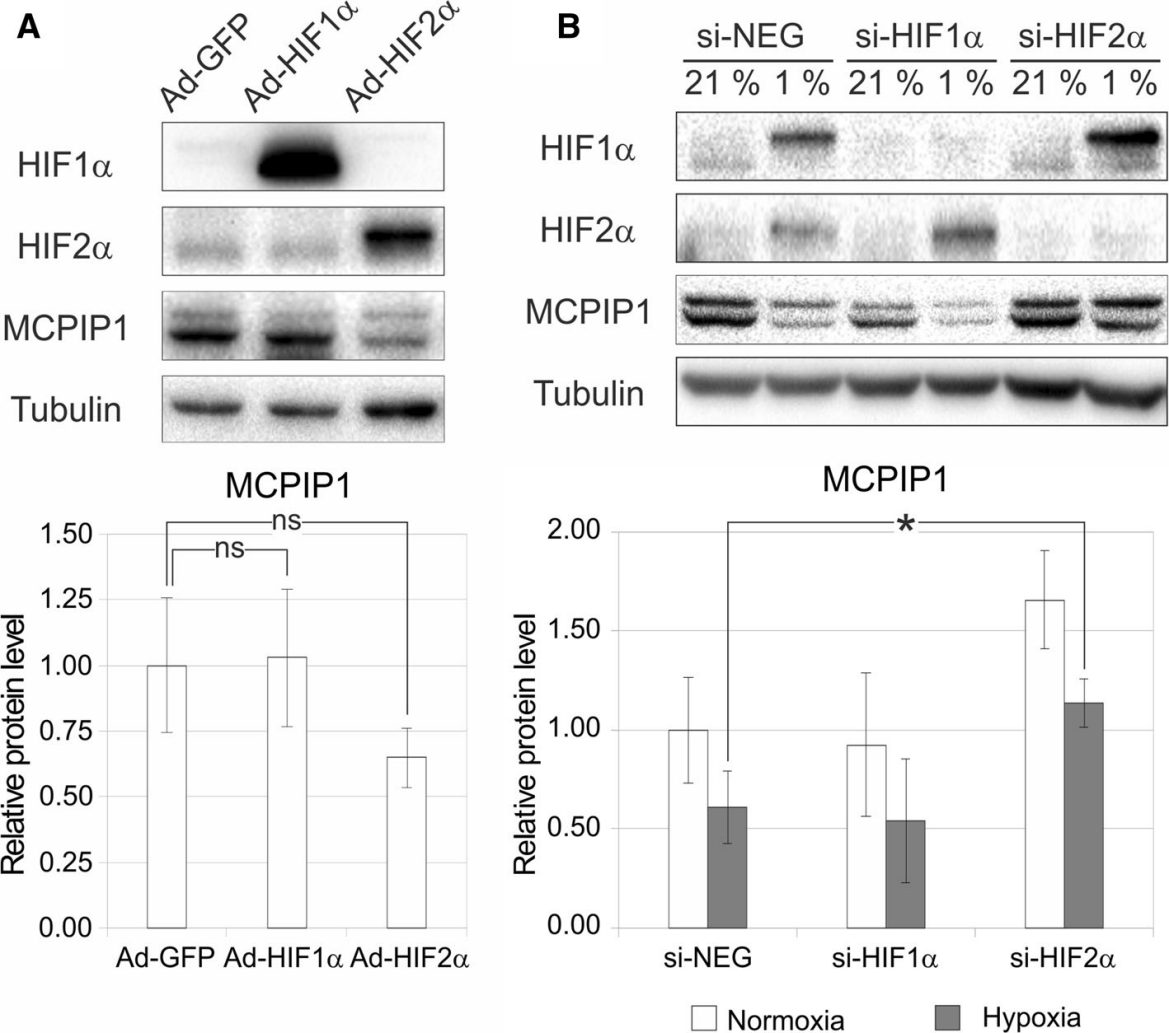
Influence of HIF1α and HIF2α overexpression (**a**) and silencing (**b**) in Caki-1 cells on MCPIP1 protein level. **a** Caki-1 cells were transduced for 24 h with adenoviral vectors containing HIF-1α or HIF-2α cDNA (Ad-HIF1α, Ad-HIF2α) or a control vector harboring green fluorescent protein (GFP) cDNA (Ad-GFP) with 50 MOI. **b** HIF1α or HIF2α expression was silenced using specific siRNAs. Control cells were transfected with negative (non-targeting) control siRNA. Cells were incubated under normoxic and hypoxic conditions for 24 h after transduction. Overexpression and silencing of HIF1α and HIF2α was confirmed by western blot. Representative immunoblots are shown. Graphs show mean ± SEM of three independent experiments. Statistical analysis was performed with ANOVA followed by Tukey’s HSD test (*p < 0.05)

In order to determine negative regulation mechanism of MCPIP1 in low oxygen supply, we analyzed how both transcription factors, HIF1α and HIF2α, influence its level. Thus, to mimic conditions present in hypoxia where both transcription factors are accumulated, Caki-1 cells were transduced with an adenoviral vector expressing HIF1α or HIF2α; then, the level of MCPIP1 was estimated by western blot. Cells transduced with an adenoviral vector expressing GFP served as an experimental control. We observed that in case of HIF2α overexpression, but not HIF1α, the level of MCPIP1 decreased (Fig. 5a, upper and lower panel). Furthermore, we examined what occurred by inhibiting expression of HIF1α and HIF2α by an siRNA strategy in cells cultured under normoxic and hypoxic conditions. We found that in case of HIF2α inhibition, but not HIF1α, and in hypoxic conditions, MCPIP1 level was increased and the change was statistically significant (Fig. 5b, upper and lower panel). Therefore, HIF2α transcription factor acts as a negative regulator of MCPIP1 expression.

### MCPIP1 overexpression in Caki-1 cells affects cell viability

As shown in Fig. 1, most human ccRCC samples have very low levels of MCPIP1 when compared to control, non-tumor tissues. Therefore, we hypothesized that diminished levels of MCPIP1 in human ccRCC samples may be a factor that promotes tumor development. To determine whether our hypothesis was correct, Caki-1 cells were transduced with a lentiviral vector expressing MCPIP1 in a doxycycline (DOX)-dependent manner (Tet-ON system; v-MCPIP1). In each experiment, Caki-1 cells transduced with an empty lentiviral vector (v-PURO) and treated with DOX served as a control. After transduction, cells were under puromycin selection (2 μg/ml) for 10 days in order to enrich the amount of cells containing vector (empty or expressing MCPIP1). Cells were then seeded in appropriate numbers on a plate in normoxic conditions, stimulated with DOX for 24 h, and finally cultured for another 24 h, in hypoxia or normoxia.

**Fig. 6:**
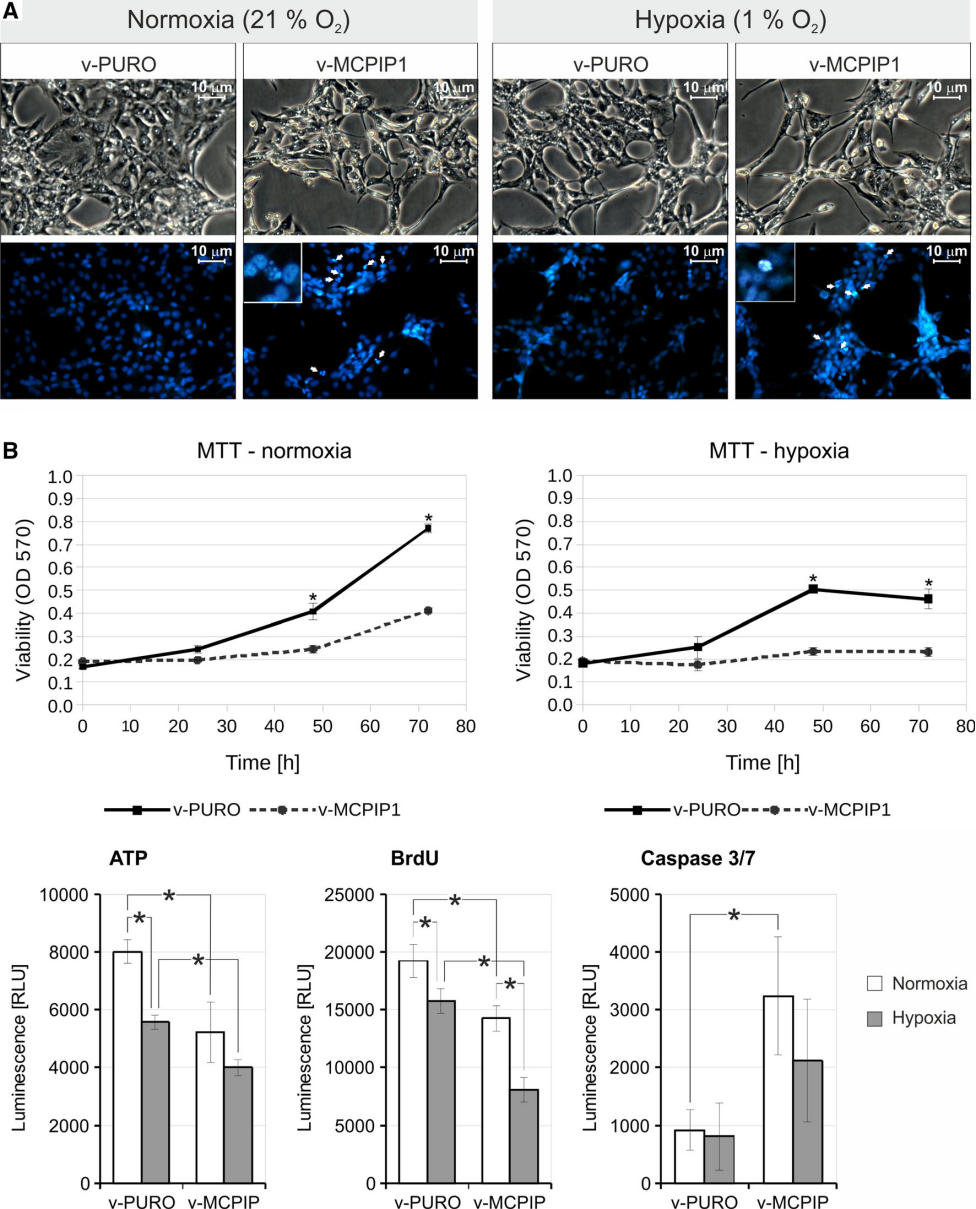
Overexpression of MCPIP1 in Caki-1 and its impact on cell biology. Cells were transduced with a Tet-ON vector overexpressing MCPIP1 (v-MCPIP1). Cells transduced with an empty vector served as a control (v-PURO). In this experiment Caki-1 cells were cultured in FBS-free medium to decrease Tet-ON system leakage. Prior to protein or mRNA isolation, cells were stimulated for 24 h with doxycycline (1 µg/ml) and then transferred to normoxic or hypoxic conditions for next 24 h. **a** Morphological changes and Hoechst 33325 staining were followed by fluorescent microscope analysis of Caki-1 cells 48 h after doxycycline induced MCPIP1 overexpression. Apoptotic cells are marked with *arrows*. **b** Viability of Caki-1 cells, proliferation rate, ATP cellular content and activity of caspase 3/7 were carried out in parallel 48 h after doxycycline addition. *Graphs* show mean ± SEM of three independent experiments. Statistical analysis was performed with ANOVA followed by Tukey’s HSD test (*p < 0.05)

Growth inhibition and changes in cellular morphology were observed 48 h after doxycycline induced MCPIP1 overexpression. We noticed that higher levels of MCPIP1 resulted in significantly lower cellular confluence in comparison with controls. Moreover, a more prominent difference was observed for cells cultured in hypoxia. Hoechst 33258 staining, followed by fluorescent microscope analysis, showed that cells expressing MCPIP1 display a nuclear morphology characteristic for late apoptosis (Fig. 6a).

To determine whether the expression of exogenous MCPIP1 changes the biology of Caki-1, we performed additional assays. The impact of MCPIP1 on cell viability was measured using an MTT assay. As shown in Fig. 6b (upper panel), cells cultured under hypoxic conditions were less viable than cells in normoxia; however, cells overexpressing MCPIP1 displayed a further significant decrease in growth under hypoxic conditions as compared to cells cultured in normoxia.

Furthermore, we observed a prominent decrease in proliferation of cells overexpressing MCPIP1, as measured by the BrdU incorporation assay. In normoxia, the proliferation rate of control cells (v-PURO) was higher than in cells overexpressing MCPIP1, while in hypoxia, the proliferation rate was generally lower than in normoxia. Control cells proliferated more efficiently than those, expressing MCPIP1(Fig. 6b, lower panel). Decreased growth was correlated with the level of ATP content which was significantly lower for cells with enhanced expression of MCPIP1. Additionally, cells with induced expression of MCPIP1 displayed activation of caspase 3/7. Cells overexpressing MCPIP1 and cultured in normoxia had 3.5 times higher caspase 3/7 activity than did control cells. In hypoxia, cells overexpressing MCPIP1 displayed stronger activation of caspase 3/7, but this difference was not statistically significant (Fig. 6b, lower panel).

### MCPIP1 strongly decreases the levels of HIFs, GLUT1, VEGFA, and IL-6 and influences the activity of signaling pathways

**Fig. 7:**
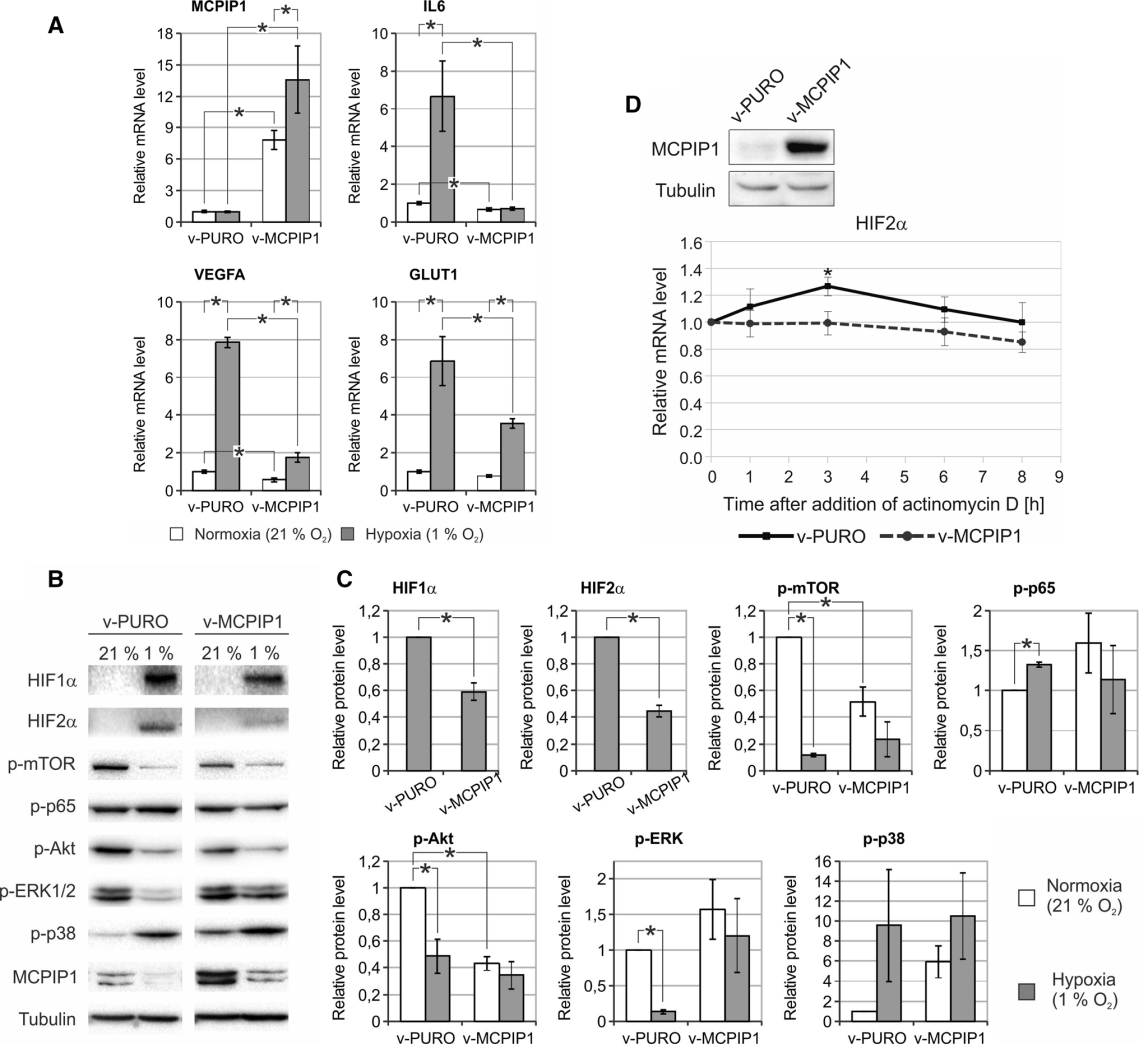
Influence of MCPIP1 overexpression on transcript level of selected genes and signaling pathways in Caki-1 cells. **a** mRNA coding for MCPIP1, VEGFA, GLUT1, and IL-6 was assessed by qRT-PCR. Results are presented as mean ± SEM of three independent experiments. For each sample transcript levels were normalized to reference gene (RPS13) expression level. The mRNA level for v-PURO Caki-1 cells in normoxia was set to 1. The *p* values were estimated using ANOVA followed by Tukey’s HSD test. Differences between cells overexpressing MCPIP1 (v-MCPIP1) and control (v-PURO) are marked (*) when statistically significant (*p* < 0.05). **b** Representative data from western blot analysis performed with specific antibodies in order to assess the phosphorylation status of selected signaling proteins. Presented data are from representative image of three independent experiments. **c** Densitometric analysis of western blots. All graphs show mean ± SEM of three independent experiments. Statistical analysis was performed with ANOVA followed by Tukey’s HSD test (*p < 0.05). **d** Caki-1 cells were transduced with a Tet-ON vector overexpressing MCPIP1 (v-MCPIP1). Cells transduced with an empty vector served as a control (v-PURO). Following 24 h stimulation with doxycycline (1 µg/ml), actinomycin D was added (5 μg/ml) for a further 1, 3, 6, and 8 h and mRNA coding for HIF2α was assessed by qRT-PCR. The transcript level was normalized to reference gene (28S rRNA) expression level. Statistical analysis was performed with ANOVA followed by Fisher’s least significant difference (LSD) test (*p < 0.05)

To further investigate the negative effects of MCPIP1 expression on cell viability, we determined the influence of MCPIP1 on genes coding for molecules that are key regulators of metabolism, angiogenesis, and inflammation: (GLUT1, VEGFA, and IL-6). In these experiments, the level of analyzed transcripts in cells transduced with an empty vector, treated with DOX and cultured in normoxia (v-PURO), was set as one. Doxycycline stimulation of MCPIP1-overexpressing cells cultured in normoxia resulted in almost eightfold higher expression of MCPIP1 transcript (v-PURO) and cultured in hypoxia, 14-fold higher expression (v-MCPIP1) as compared to controls (Fig. 7a). As suspected, hypoxia significantly induces the expression of *GLUT1*, *VEGFA*, and *IL*-*6* transcripts in control cells. Overexpression of MCPIP1 leads to a strong decrease of the level of transcripts coding for those proteins in normoxia and in hypoxia; however, the effect in hypoxic conditions is especially meaningful (Fig. 7a).

In the next step, we determined the activation of selected signaling proteins that are important in tumor growth. We examined how enhanced expression of MCPIP1 influences the activation status of HIFs, NF-κB, p38, ERK1/2, Akt, and mTOR. Although the level of endogenous (v-PURO) and exogenous MCPIP1 (v-MCPIP1) was generally lower in cells cultured in hypoxia than in normoxia, the changes in activity of the mentioned proteins were observed after MCPIP1 overexpression.

The most prominent changes were observed for HIFs. First of all, hypoxia strongly activates accumulation of HIFs as both proteins were not detectable in cells cultured in normoxic conditions. Interestingly, the cells overexpressing MCPIP1 and cultured in hypoxia exhibit a significantly lower level of HIF1α and HIF2α in comparison with the cells transduced with an empty vector. Furthermore, overexpression of MCPIP1 (v-MCPIP1) in normoxic conditions results in the lower activation of mTOR and protein kinase B (Akt) and higher activation of ERK1/2 and p-38, and p-65 (subunit of NF-κB), although in case Akt, ERK1/2, and p-65 changes are not statistically significant. Moreover, in hypoxic conditions, cells overexpressing MCPIP1 display slightly increased phosphorylation of mTOR and ERK1/2 although changes were not statistically significant. In case of Akt and p-65 molecules, a decreasing trend was observed, but these differences were also not statistically significant (Fig. 7b, c).

MCPIP1 acts as an endonuclease controlling stability of many transcripts; therefore, we ask the question whether diminished level of HIF2α might be the result of regulation of HIF2α transcript stability by MCPIP1. We carried out experiment where cells overexpressing MCPIP1 were treated with actinomycin D and then, total RNA was isolated in selected time points, and Q-RT-PCR was done. We found that cells overexpressing MCPIP1 display shorter half time of transcript coding for HIF2, thus MCPIP1 regulates HIF2α on transcript level (Fig. 7d).

## Discussion

In this study, we found that MCPIP1 levels are substantially lower in ccRCC samples than in the surrounding tissues. Using Caki-1 cell line as a model, we found that proteasome regulates MCPIP1 expression; however, in the cells cultured under hypoxic conditions, the accumulation of MCPIP1 is not so efficient as it is in the case of cells kept under normal oxygen supply. We showed that one of the factors negatively regulating MCPIP1 during hypoxic conditions is HIF2α; however, the exact mechanism is still unknown. Furthermore, we also found that MCPIP1 negatively regulates the levels of HIFs and that in the case of HIF2α, the mechanism is based on the shortening of the half-life of its transcript coding for this protein. Negative regulation of HIF2α by MCPIP1 may explain, at least partially, some features of ccRCC development, including proinflammatory and proangiogenic changes. MCPIP1 overexpression strongly reduces transcripts coding for *VEGFA*, *IL*-*6*, and *GLUT1*, especially in hypoxic conditions. Additionally, MCPIP1 decreases phosphorylation of mTOR and Akt in normoxic conditions and, finally, decreases proliferation rate of Caki-1 cells and activates caspases 3/7.

Current treatment for ccRCC is based on nephron-sparing and minimally invasive surgery, which is usually considered for tumors smaller than 4 cm in diameter (stage pT1a), while radical nephrectomy is used for tumors larger than 4 cm. Despite increased detection of early cases, approximately 50% of patients display metastatic disease [21, 22]. Current therapies are based on the treatment with immunomodulatory drugs [23], multiple tyrosine kinase receptor inhibitors [24–26], inhibitors of mammalian target of rapamycin [27], or dendritic cell (DC)-based vaccines [28, 29]. Targeted therapies have markedly improved patient outcomes, but they rarely induce complete responses, and the major problem of such therapies is resistance to treatment. This work provides a strong rationale for testing novel agents to improve ccRCC therapy.

MCPIP1 plays a key role in the regulation of inflammatory processes via controlling the levels of transcripts coding for inflammation-related modulators but also proteins involved in many other biological processes [8, 10, 12, 13, 30]. The impact of inflammation on cancer development was shown as early as 1863 by Virchow [31]. Many subsequent studies confirmed that tumor microenvironment is often infiltrated by innate and adaptive immune system cells, releasing growth factors, proangiogenic factors and extracellular matrix (ECM)-modifying enzymes. All these factors facilitate angiogenesis while promote growth, invasion, and metastasis of tumor cells.

We observed that the levels of MCPIP1 transcript and protein were diminished in the vast majority of ccRCC tissues. For subsequent studies, to investigate the mechanism of MCPIP1 downregulation in ccRCC samples, we used Caki-1 cell line as a model. These cells possess an active form of VHL and are characterized by significantly lower level of MCPIP1 in comparison with the A498 cell line, which features an inactive form of VHL. This protein is responsible for assigning other proteins for proteasomal degradation and has a well-documented role in ccRCC development [32, 33]. Furthermore, somatic *VHL* mutations are detected in 60–90% of patients with ccRCC. Aberrations in *VHL* gene function either through mutation or promoter hypermethylation [34, 35]. Our experiments performed in both Caki-1 and A498 cells transfected with wild-type or mutated pVHL indicated that this protein is not involved in MCPIP1 regulation. The fact that MCPIP1 is diminished on both transcript and protein levels in most ccRCC cases is additional evidence that in these cells a VHL-independent mechanism that is responsible for downregulation of this RNase exists.

Our previous studies on HepG2 and HeLa cells indicated that inhibition of proteasome resulted in the accumulation of MCPIP1 and that this upregulation of MCPIP1 in MG132 treated cells is a result of *de novo* synthesis [12, 19]. Similar phenomenon was observed in Caki-1 cells after treatment with MG132, in both hypoxic and normoxic conditions. Administration of proteasome inhibitor to Caki-1 cell culture medium led to the increase of MCPIP1 in normal and low oxygen supply conditions, although, in hypoxia, the effect was less prominent than in normoxia. The fact that MCPIP1 is increased in MG132-treated cells may be explained by enhanced activation of NF-κB and p38 MAP kinase. Both proteins were shown to be important in proteasome dependent MCPIP1 gene upregulation [12, 19]. A strong accumulation of MCPIP1 after proteasome inhibition in Caki-1 cells is the result of *de novo* synthesis, as the experiment with addition of cycloheximide and MG132 indicated. The fact that this synthesis was not so efficient in hypoxic conditions means that hypoxia activates factor(s) that negatively regulate MCPIP1 expression.

Under hypoxic conditions, MCPIP1 is strongly downregulated on transcript and protein levels. This phenomenon is observed for ccRCC cell line with active (Caki-1) and inactive (A498) VHL form. Furthermore, the same effects were observed for non-cancer cells: human embryonic kidney cells (HEK 293) and an immortalized proximal tubule epithelial *cell line* from normal adult human kidney (HK-2). The fact that MCPIP1 is downregulated under hypoxic conditions has an important impact on many cellular processes. MCPIP1 influences the levels of many transcripts coding for proinflammatory cytokines (IL-6, IL-1, and many others), transcription factors (NFkappaB, C/EBPbeta), signaling proteins (JNK, Akt/mTor) [8–13, 36–38]. The whole list of targets is not known yet; thus, it is difficult to predict which other cellular processes are altered by diminished level of MCPIP1.

To investigate the mechanism involved in a negative regulation of MCPIP1 in hypoxic conditions in both control cells and those treated with MG132, we tested the role of HIFs in this process. Numerous studies have shown that HIFs are crucial in tumor development [39–43]. Importantly, both HIF isoforms may independently regulate gene expression, e.g., in human endothelial cells, IL-8 expression is decreased by HIF-1α, whereas HIF-2α overexpression leads to the induction of IL-8 mRNA and protein level; however, of the HIF subunits, HIF-2α appears to be more oncogenic than HIF-1α activating pro-tumorigenic target genes. In addition, recent studies indicated that HIF-1α, more than HIF-2α, can undergo proteasomal degradation in VHL -/- RCC cells, thus HIF-2α is an important player in ccRCC development [44, 45]. It is also worth noting here that using MDR and logistic regression methods, we discovered a complex network of interactions involving six genes implicated in RCC (*SCARB1*, *GNAS1*, *BIRC5*, *EPAS1*, *VDR* and *MC1R*), of which the *EPAS1* gene encodes HIF2α [46]. Using Caki -1 cells as a model, for the first time we have shown that HIF2α, but not HIF1α, is a key player in the downregulation of MCPIP1 transcript and protein, particularly in hypoxia. HIF-1 and HIF-2, despite some overlapping effects, can uniquely regulate distinct genes. HIF2α may regulate gene expression contributing to various stages of MCPIP1 downregulation by: binding directly to specific binding sites present in the regulatory regions of target genes and activating gene expression or stimulating miRNA expression that will affect target gene expression. The evidence for the latter is derived from the studies of the influence of MCPIP1 overexpression on Caki-1 cell viability. In spite of MCPIP1 expression from exogenous template (a vector overexpressing MCPIP1), the level of this protein was diminished in hypoxia. Vector regulatory sequences do not contain sequences recognized by HIF2α; thus, it is possible that regulation of MCPIP1 expression is triggered by some miRNAs regulated by HIF2α activated in hypoxia. It can be with an agreement what we observe in the experiment where MCPIP1 transcript level is analyzed in Caki-1 cells kept in hypoxic conditions for 12 and 24 h. The level of MCPIP1 mRNA is higher after 12 h than after 24 h. Following miRNA-dependent hypothesis of MCPIP1 regulation, one can imagine that after induction of hypoxia HIF2α is activated. Then, this transcription factor induces expression of specific miRNA that regulates MCPIP1 transcript. As a result, MCPIP1 transcript level is lower after 24 h than after 12 h, because miRNA continues degradation in cell cultured upon hypoxic conditions. However, the exact mechanism of this regulation deserves further investigation.

Under normoxic conditions, overexpressed MCPIP1 significantly reduces mTOR and Akt activity, while changes in hypoxia are almost not detectable. In case of mTOR, there is a delicate tendency to upregulation, although the difference is not statistically significant. From the other hand, phosphorylation of both proteins in ccRCC samples is enhanced; therefore, this phenomenon might be at least partially the result of diminished level of MCPIP1 in this tumor. At the end, it must enhance proliferation rate of tumor cells. Besides having an influence on mTOR and Akt, overexpressed MCPIP1 displays a tendency to a higher levels of the phosphorylated forms of MAP kinases (p38 and ERK1/2) in the same conditions, while in hypoxia only slightly stronger phosphorylation of ERK1/2 was observed, without influence on p38 kinase. It is well known that both kinases are activated by stress. In our experimental conditions, cells were exposed to stress induced by hypoxia and by overexpression of MCPIP1. Both kinases are important in response to hypoxic shift and cells adaptation to oxygen deprivation [47]. In the case of MCPIP1, there are evidences that it induces production of reactive oxygen species (ROS), what results in the generation of cellular stress [48]. MCPIP1 does not change phosphorylation status of NF-κB subunit, p65. It is possible that the effect of MCPIP1 on selected signaling pathways would be more meaningful under hypoxic conditions in case of higher level of this protein. Unfortunately, MCPIP1 is diminished in hypoxia, even after overexpression, and further studies are essential to understand this mechanism.

For the first time, we report that MCPIP1 negatively regulates HIF1α and HIF2α level under hypoxic conditions. Conversely, one potential reason is that cells overexpressing MCPIP1 display lower expression of transcripts coding for *GLUT1* and *VEGFA*, which is the result of HIFs negative regulation by MCPIP1. As already mentioned, there are many evidences that MCPIP1 acts as an RNase controlling stability of transcripts. Direct regulation of IL-6 by MCPIP1 is already well described [10]. Here we show for the first time that MCPIP1 controls half-life of transcript coding for HIF2α but not for VEGFA. It is possible that downregulation of *VEGFA* transcript might be the effect of negative regulation of HIF2α by MCPIP1. On the basis of these observations, we can assume that the prominent vascularization observed in ccRCC samples might be explained by diminished level of MCPIP1 in this tumor. Besides HIFs, also IL-6 exhibits proangiogenic properties acting as an inductor of VEGFA in a STAT3 and HIF-dependent manner [49–52]. IL-6 may also directly influence blood vessel formation by promoting endothelial progenitor cell migration and proliferation [53, 54], and/or the stimulation of vascular smooth muscle cell (VSMC) migration [55]. Interestingly, Baldewijns and coauthors found no significant association between VHL mutation or methylation and angiogenesis/tumor parameters [56]. It is possible that MCPIP1 is a missing link, because its expression and stability is not dependent on VHL but has a significant influence on the level of proangiogenic factors.

Consequently, decreased MCPIP1 levels in ccRCC may promote tumor development and metastasis. Additionally, our results clearly indicate that MCPIP1 also influences cell metabolism, by negative regulation of *GLUT1* transcript. Strong, MCPIP1-dependent downregulation of HIFs correlates with worsened cell survival, proliferation rate, ATP content and activation of caspases 3/7. All these changes triggered by MCPIP1 in Caki-1 cells are potentially important in tumor biology, making it plausible that this protein is important for consideration as a therapeutic target in ccRCC.

Negative roles of MCPIP1 in cell proliferation and viability were already described in different cancer cell types. Overexpression of MCPIP1 modulates transcriptome including miRNAs, in human neuroblastoma cells [57]. Furthermore, it was also shown that MCPIP1 functions as a potent tumor suppressor and induces apoptosis of breast tumor cells by selectively enhancing mRNA decay of antiapoptotic gene transcripts, including *Bcl2L1, Bcl2A1, RelB, Birc3*, and *Bcl3* [58]. It is possible that MCPIP1 influences several cellular processes acting as an RNase and regulating stability of different important RNA molecules. However, regardless of the type of cellular process in which MCPIP1 is involved, its significance in antitumor response seems to be undisputed.

**Fig. 8:**
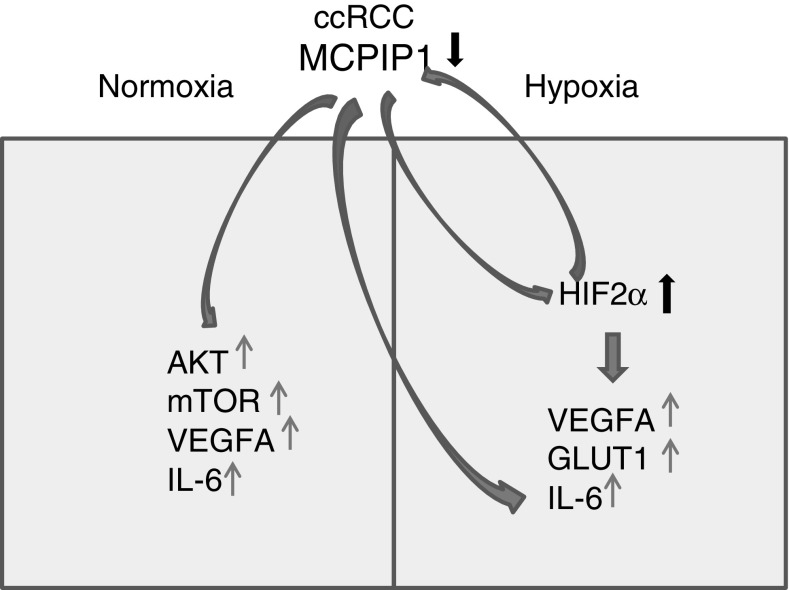
Role of MCPIP1 in ccRCC development—detailed description in the “Discussion” section

In conclusions, MCPIP1 is downregulated in ccRCC samples on mRNA and protein levels. On the basis of our result, we can assume that diminished level of MCPIP1 in ccRCC will elevate proliferation rate increasing activity of AKT and mTOR in normoxic conditions, decrease activation of caspase 3/7, upregulate HIFs and, furthermore, will decrease the levels of transcripts coding for proangiogenic, proinflammatory and metabolic regulators: VEGFA, IL-6 and GLUT1. Altogether, diminished level of MCPIP1 observed in ccRCC might be a driving wheel, enhancing proliferative rate, metabolism and angiogenesis of ccRCC (Fig. 8).

## Materials and methods

### Patient tissue samples

Normal and tumor renal tissues pairs were obtained from patients surgically treated for renal cancer in Oncology Center in Krakow under the supervision of the local ethical committee (approval no 68/KBL/OIL/2011). Samples were subjected to histological evaluation by pathologists and diagnosed according to criteria set forth by World Health Organization classification. In each case, the sample was divided into two pieces, with one frozen at −80 °C (for protein isolation), and the other was incubated overnight in RNA-later (Invitrogen) at 4 °C and then stored in −80 C to be used for RNA isolation.

### Cell culture

Human clear cell renal cell carcinoma cell lines, Caki-1 and A498, were cultured in Eagle’s minimal essential medium (EMEM) (Lonza) containing 10% fetal bovine serum (FBS), (BioWest SAS, Nuaillé, France) at 37 °C in a 5% CO_2_ atmosphere. Cells were passaged three times a week, at 80–90% confluence.

### Transfection/transduction of Caki-1

For stable expression of MCPIP1, doxycycline-dependent overexpression Tet-ON system was used. Caki-1 cells were seeded on 6-well plates at 50–70% confluency (3 × 10^5^ cells per well). Lentivirus was added at MOI-50. Cells were incubated with viruses for 24 h, and then medium was replaced. After an additional 24 h, puromycin was added at concentration 2 µg/ml and selection of stably transduced cells was carried out for next 10 days. Stimulation of MCPIP1 expression was performed in FBS-free medium (DMEM/F12 supplemented with BSA (0.5 mg/ml), transferrin (5 μg/ml) and Na_2_SeO_3_ (2 ng/ml)) to decrease Tet-ON system leakage.

Caki-1 cells at 50–70% confluence were transfected with wild-type VHL or its mutant form del-VHL (a deletion of C-terminal domain) (both vectors were kind gift of Professor Sánchez-Prieto R [14]) using Lipofectamine 2000. Procedure was performed according to manufacturer’s recommendation. For 6-well plate, 3 × 10^5^ cells were seeded in 2 ml and the following day, the transfection was performed. Briefly 7.5 µl of Lipofectamine 2000 was diluted in 100 μl of EMEM without FBS and 2.5 µg DNA (p-VHL vector, p-mVHL or empty vector) was diluted in 100 µl EMEM without FBS. After 5 min, diluted plasmid DNA and Lipofectamine 2000 were combined and incubated 15 min at room temperature. DNA-lipofectamine complexes were then added to cells and mixed gently by rocking. To reduce toxicity, cell medium was changed 5 h after transfection and the cells were incubated for 24–72 h for further experiments.

Adenoviral vectors containing HIF1α or HIF2α cDNA (Ad-HIF1α, Ad-HIF2α) were a kind gift from Prof. Seppo Yla-Herttuala (Kuopio, Finland) and Prof. Lorenz Poellinger (Stockholm, Sweden). The pAdHIF-1α was generated as described previously [59]. Briefly, the construct was stabilized against prolyl hydroxylation and subsequent ubiquitin-mediated proteolytic degradation in normoxic conditions by point mutations (P402A/P563A). A control vector harboring green fluorescent protein (GFP) cDNA (AdGFP) was produced using the AdenoX system as described previously [59].

### Transfection with small interfering RNA (siRNA)

Cells were transfected with 25 nM of chemically synthesized siRNA targeted against human HIF1α or HIF2α mRNA. As a control scrambled siRNA was used. Briefly, cells were plated into 6-well plates 1 day prior to transfection to obtain 50–70% confluency. siRNA and Lipofectamine 2000 were separately diluted in Opti-MEM without serum, incubated 5 min at room temperature, combined, and then incubated for 20 min at room temperature. Forty-eight hours post-transfection protein cell lysates were prepared and used for further analysis.

### Hypoxia

Caki-1 cells were seeded on 12-well plate (1.2 × 10^5^ cells/well) at approximate 70% confluency. Following 24-h incubation, plates were placed in cell culture incubators with hypoxic condition (1% O_2_) or in atmospheric conditions (21% O_2_). Protein and RNA were isolated after 12 and 24 h of incubation.

### RNA isolation

Total RNA was isolated using the guanidium isothiocyanate (GTC) method. The integrity of ribosomal RNA and DNA contamination was examined using denaturing formaldehyde gel electrophoresis (l%). Protein and phenol contamination and concentration of total RNA was assessed by determining the ratio A260/280 and A260/230, respectively (NanoDrop).

### qRT-PCR

For the real-time PCR, 1 μg of total RNA was reverse-transcribed using the oligo(dT) primer (Promega), random hexamers (EURx), and M-MLV reverse transcriptase (Promega). Following synthesis, cDNA was diluted fivefold and real-time PCR was carried out using the Eco Real-Time PCR System (Illumina) and a Sybr Green-based master mix (A&A Biotechnology). For tissue samples peptidylprolyl isomerase A (PPIA) was used as a reference gene. For cell cultures, each sample was normalized to reference gene ribosomal protein S13 (RPS13). For mRNA half-life analysis as a reference gene 28S ribosomal RNA was used (28S rRNA). The relative level of transcripts was quantified by the ΔΔCT method.

Q-RT-PCR was carried out for MCPIP1 transcript with primers: ggaagcagccgtgtccctatg and tccaggctgcactgctcactc (NM_025079.2); for IL-6 transcript with primers: ggtacatcctcgacggcatct and gtgcctctttgctgctttcac; for RPS13 with primers: tcggctttaccctatcgacgcag and acgtacttgtgcaacaccatgtga; for VEGFA transcript with primers: agaaaatccctgtgggccttgctc and gcctcggcttgtcacatctgcaa; for SLC2A1(GLUT1) transcript with primers: tccctgcagtttggctacaa and gcaggatgctctccccatag; for CXCL8 (IL-8) with primers: atgacttccaagctggccgtggct and atgacttccaagctggccgtggct, for HIF1α with primers: caagaacctactgctaatgc and ttatgtatgtgggtaggagatg; for EPAS1(HIF2α) with primers: gcgctagactccgagaacat and tggccacttactacctgaccctt, for 28S rRNA with primers: cacccactaatagggaacgtg and ctgacttagaggcgttcagtc.

### HIF2α mRNA half-life analysis

Caki-1 cells with a stable expression of MCPIP1 (Tet-ON system vectors) were seeded on 12-well plate (1.2 × 10^5^ cells/well). After 24-h incubation, actinomycin D (BioShop) was added at a final concentration of 5 mg/ml. Total RNA was collected at the following time points: 1, 3, 6, and 8 h. After cDNA synthesis, Q-RT-PCR was carried out as described above.

### Protein isolation

After washing with ice-cold PBS, cells were harvested and lysed on ice in RIPA buffer (25 mM Tris–HCl, pH 7.6, 150 mM NaCl, 1% NP-40, 1% sodium deoxycholate, 0.1% SDS) supplemented with Protease Inhibitor Cocktail (Sigma) and PhosSTOP Phosphatase Inhibitor Cocktail (Roche). The protein concentration in cell lysates was measured with the bicinchoninic acid assay.

### Western blotting

Cell lysates were separated by SDS/PAGE (8, 10 or 12% polyacrylamide gel), electrotransfered to PVDF membrane (Millipore,) and blocked for 1 h (at room temperature) in 5% non-fat powdered milk dissolved in Tris-buffered saline containing 0.1% Tween 20 (BioShop, Burlington, Canada). Membranes were incubated with primary antibody overnight at 4 °C. After incubation with secondary antibody for 1 h, room temperature, chemiluminescence was detected using Immobilon Western HRP substrate (Millipore) with ChemiDoc system (BioRad). The following antibodies and dilutions were used: rabbit anti-MCPIP1 (1:1000, GeneTex), HIF1α (1:1000), HIF2α (1:1000), VHL (1:500), Akt (1:1000), Phospho-Akt (1:2000), SAPK/JNK (1:500), Phospho-SAPK/JNK (1:1000), p38 (1:1000), Phospho-p38 (1:1000), ERK1/2 (1:1000), Phospho-ERK1/2 (1:1000). All mentioned antibodies were from Cell Signaling Technology. Tubulin (1:4000, Calbiochem; Merck Millipore, Billerica, MA, USA) or in case tissue samples, GAPDH (1:30,000 Sigma-Aldrich) antibodies were used as a loading control. The following secondary antibodies were used: peroxidase-conjugated anti-rabbit IgG (1:30,000; Sigma) and peroxidase-conjugated anti-mouse IgG (1:10,000, Sigma).

### ATP content assay

Cell viability was analyzed with ATPLite—Luminescence ATP Detection Assay System (PerkinElmer). Cells were plated on 96-well white plates (5 × 10^3^ per well), and after 24 h, MCPIP1 expression was stimulated with doxycycline. Quantification of intracellular ATP content was carried out according to the manufacturer’s instructions after 48 h of exposure. The luminescence was measured using the Tecan Spectra Fluor Plus Microplate Reader (Tecan Group Ltd., Männedorf, Switzerland).

### BrdU assay

Cell proliferation was measured with BrdU incorporation assay using the chemiluminescent BrdU Cell Proliferation Assay Kit (Roche) as it was performed [60]. Chemiluminescence was measured using the Tecan Spectra Fluor Plus Microplate Reader (Tecan Group Ltd.) in three independent experiments, each performed in triplicate.

### MTT assay

Cell viability was measured using the colorimetric MTT assay as described [54]. Absorbance was measured using the Tecan Spectra Fluor Plus Microplate Reader (Tecan Group Ltd.) at 570 nm with the reference wavelength of 500 nm. Three independent experiments were performed, each in quintuplicate.

### Statistical analysis

All statistical analysis was performed using Statistica 10 (Statsoft). The statistical significance for differences between normal and tumor tissue samples was assessed by the Mann–Whitney *U* test. Experiments were conducted in three independent repetitions. The standard error of the mean (SEM) is presented on the graphs. Testing of multiple samples was performed with ANOVA followed by Tukey’s HSD (honest significant difference) test. The *p* values were marked with the asterisks on the charts (*p < 0.05).

## Electronic supplementary material

Below is the link to the electronic supplementary material.

Supplementary Fig. 1. Differences in the expression level of selected markers between Caki-1and A498 cells. Cells were seeded on 30 mm cell culture dishes and 24 h later protein and totalmRNA were isolated. For each sample transcript level was normalized to reference gene (RPS13)expression level. (A) The mRNA level for Caki-1 cells was set to 1. Each bar represents themean ± SEM of three independent experiments. (B) The protein level detected by western blot andrepresentative images are shown. The mean value for the Caki-1 was set as 1 and *p*-values wereestimated using Mann-Whitney (Wilcoxon) W-test (*p < 0.05) (JPEG 700 kb)

Supplementary Fig. 2. Reintroduction of pVHL in A498 cells. Cells (EMEM + 10% FBS) weretransfected with plasmid HA-VHL-pRc/CMV containing wild type VHL gene or HA-VHL 1-167-pRc/CMV plasmid containing mutated VHL gene (deletion of C-terminal domain, del-VHL). As acontrol, untreated cells (NT) and cells transfected with empty pcDNA3 plasmid (pcDNA) wereused. (A) After 24 h a western blot was performed with specific antibodies for MCPIP1, VHLand α-tubulin. (B) mRNA coding for VEGFA and GLUT1 was assessed by qRT-PCR. The transcriptlevel was normalized to reference gene (RPS13) expression level. Each bar represents themean ± SEM of three independent experiments. Statistical analysis was performed with ANOVAfollowed by Tukey’s HSD test (*, *p* < 0.05) (JPEG 485 kb)

Supplementary Fig. 3. Influence of hypoxia on MCPIP1 protein and mRNA levels. (A) A498 cells were seeded on 30 mm cell culture dishes (under normoxic and hypoxicconditions. Protein and total mRNA were isolated after 12 and 24 h. qRT-PCR was performed andthe transcript level was normalized to reference gene (RPS13). The level of mRNA from cells keptin normoxia was set to 1. Protein levels were detected by western blot. (B) HEK293 (cultured inDMEM + 10% FBS) and Caki -2 (cultured in McCoy’s-5A + 10% FBS) cells were cultured for 12 hunder normoxic and hypoxic conditions. (C) HK-2 and Caki-1 (for HK-2 DMEM+10%FBS wereused) were seeded on 6-well plate. After 24 h cells were cultured for another 24 h in normoxic andhypoxic conditions. Protein level for MCPIP1 was estimated by western blot. Representativeimages are shown from three independent experiments. Statistical analysis was performed withANOVA followed by Tukey’s HSD test (JPEG 367 kb)

Supplementary material 4 (JPEG 261 kb)
