# Supplementary figures and images for: MCPIP1 contributes to clear cell renal cell carcinomas development
Source: Angiogenesis. 2017 Feb 14;20(3):325–40. doi: 10.1007/s10456-017-9540-2 (PMC5511613; doi:10.1007/s10456-017-9540-2)

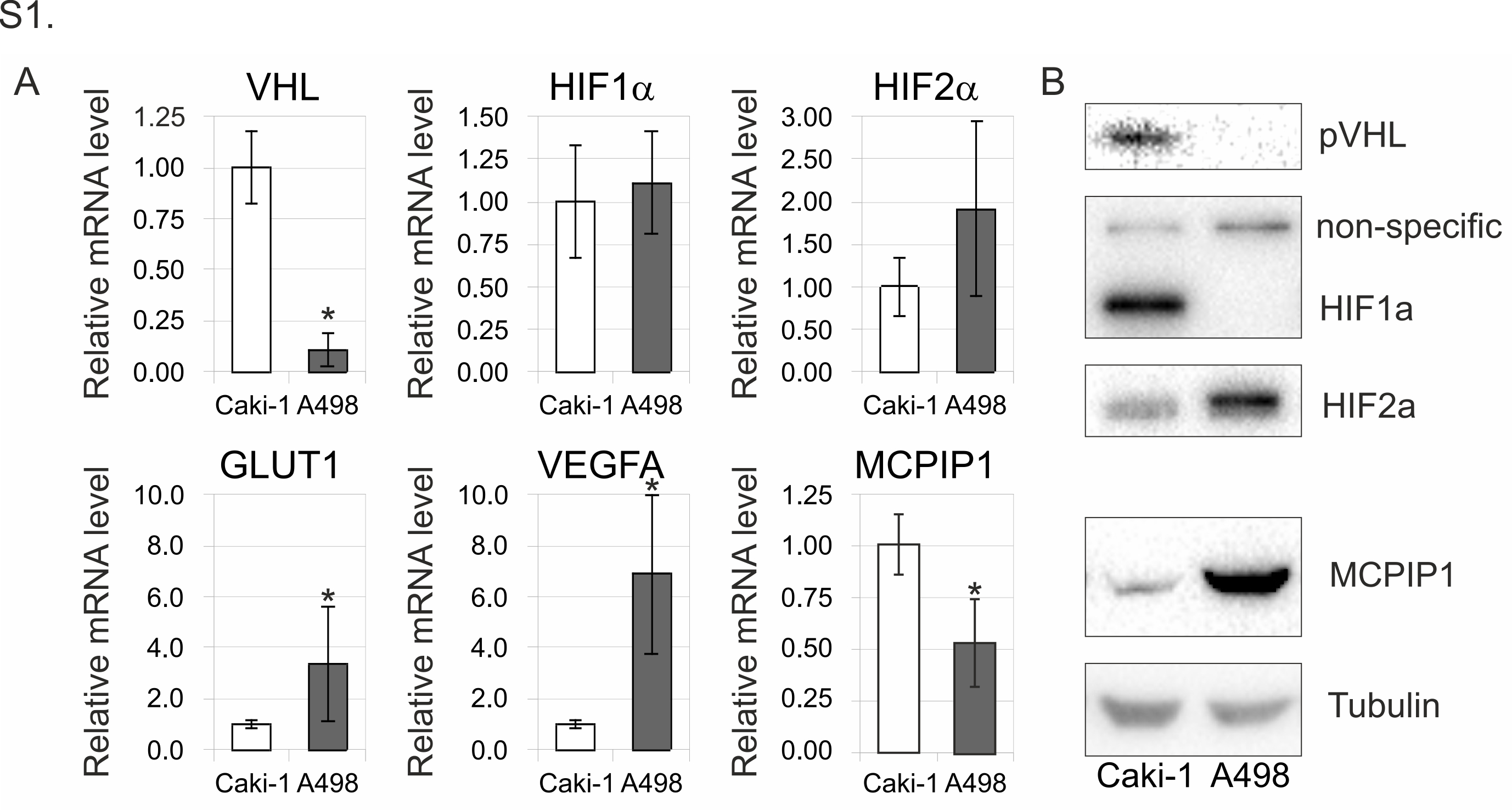

Supplement: Supplementary file 1 — Supplementary Fig. 1. Differences in the expression level of selected markers between Caki-1and A498 cells. Cells were seeded on 30 mm cell culture dishes and 24 h later protein and totalmRNA were isolated. For each sample transcript level was normalized to reference gene (RPS13)expression level. (A) The mRNA level for Caki-1 cells was set to 1. Each bar represents themean ± SEM of three independent experiments. (B) The protein level detected by western blot andrepresentative images are shown. The mean value for the Caki-1 was set as 1 and p-values wereestimated using Mann-Whitney (Wilcoxon) W-test (*p < 0.05) (JPEG 700 kb) [file 10456_2017_9540_MOESM1_ESM.jpg]

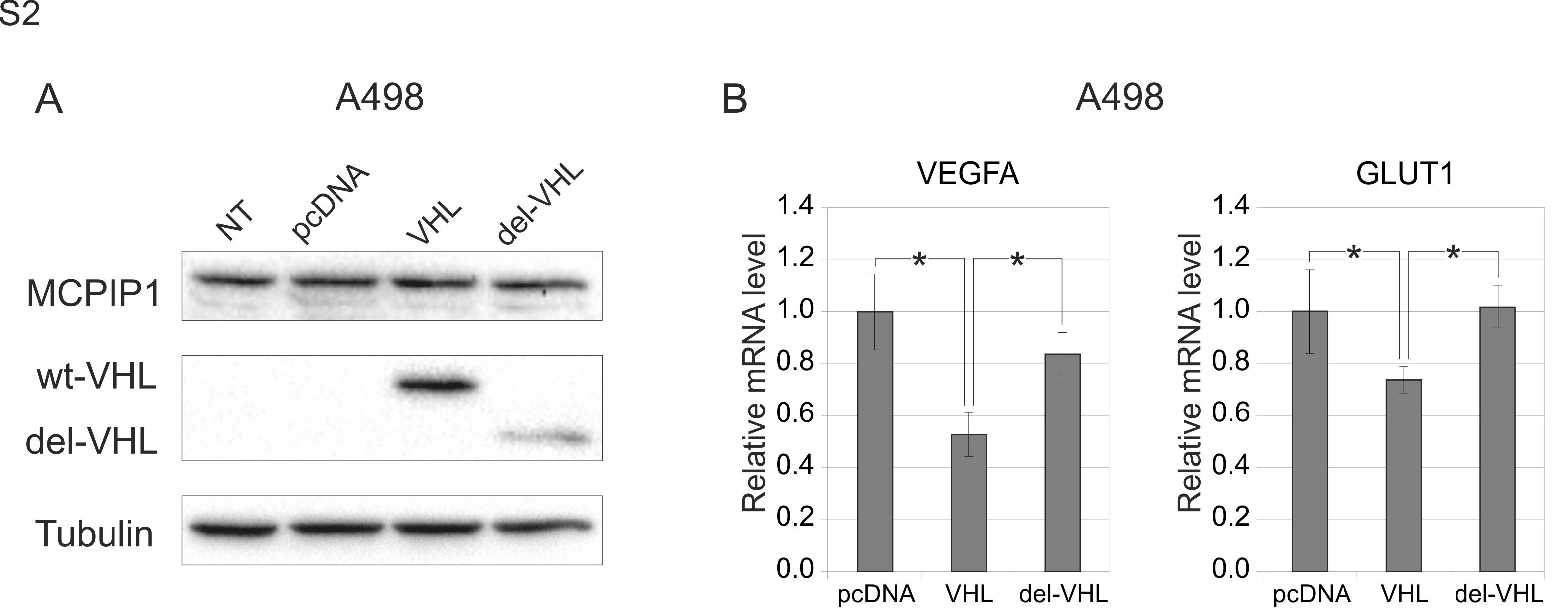

Supplement: Supplementary file 2 — Supplementary Fig. 2. Reintroduction of pVHL in A498 cells. Cells (EMEM + 10% FBS) weretransfected with plasmid HA-VHL-pRc/CMV containing wild type VHL gene or HA-VHL 1-167-pRc/CMV plasmid containing mutated VHL gene (deletion of C-terminal domain, del-VHL). As acontrol, untreated cells (NT) and cells transfected with empty pcDNA3 plasmid (pcDNA) wereused. (A) After 24 h a western blot was performed with specific antibodies for MCPIP1, VHLand α-tubulin. (B) mRNA coding for VEGFA and GLUT1 was assessed by qRT-PCR. The transcriptlevel was normalized to reference gene (RPS13) expression level. Each bar represents themean ± SEM of three independent experiments. Statistical analysis was performed with ANOVAfollowed by Tukey’s HSD test (*, p < 0.05) (JPEG 485 kb) [file 10456_2017_9540_MOESM2_ESM.jpg]

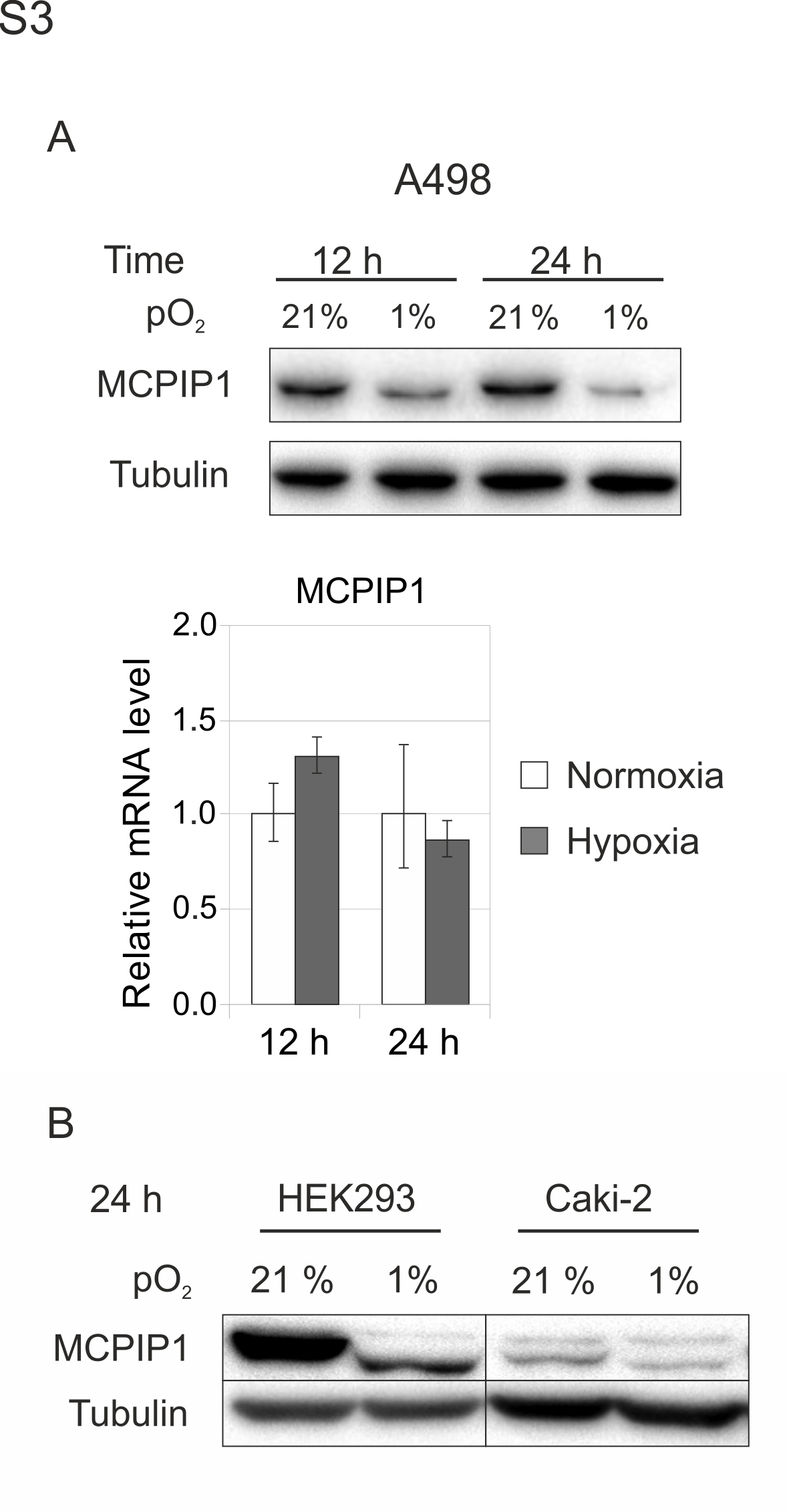

Supplement: Supplementary file 3 — Supplementary Fig. 3. Influence of hypoxia on MCPIP1 protein and mRNA levels. (A) A498 cells were seeded on 30 mm cell culture dishes (under normoxic and hypoxicconditions. Protein and total mRNA were isolated after 12 and 24 h. qRT-PCR was performed andthe transcript level was normalized to reference gene (RPS13). The level of mRNA from cells keptin normoxia was set to 1. Protein levels were detected by western blot. (B) HEK293 (cultured inDMEM + 10% FBS) and Caki -2 (cultured in McCoy’s-5A + 10% FBS) cells were cultured for 12 hunder normoxic and hypoxic conditions. (C) HK-2 and Caki-1 (for HK-2 DMEM+10%FBS wereused) were seeded on 6-well plate. After 24 h cells were cultured for another 24 h in normoxic andhypoxic conditions. Protein level for MCPIP1 was estimated by western blot. Representativeimages are shown from three independent experiments. Statistical analysis was performed withANOVA followed by Tukey’s HSD test (JPEG 367 kb) [file 10456_2017_9540_MOESM3_ESM.jpg]

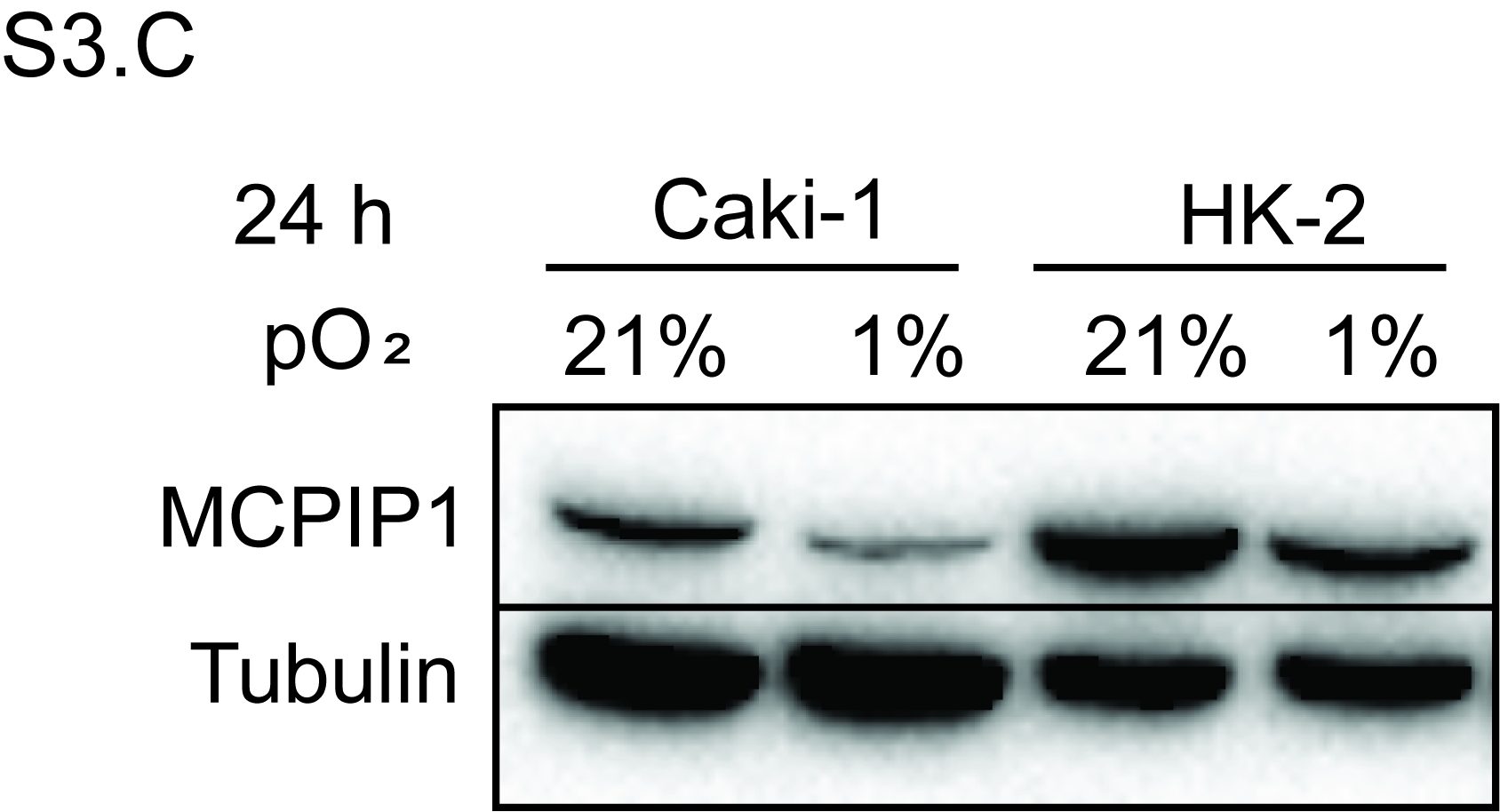

Supplement: Supplementary file 4 — Supplementary material 4 (JPEG 261 kb) [file 10456_2017_9540_MOESM4_ESM.jpg]
